# RNA-seq analysis reveals transcriptome changes in livers from *Efcab4b* knockout mice

**DOI:** 10.1016/j.bbrep.2025.101944

**Published:** 2025-02-15

**Authors:** Chew W. Cheng, Lucia Pedicini, Cintli Morales Alcala, Fenia Deligianni, Jessica Smith, Ryan D. Murray, Harriet J. Todd, Niamh Forde, Lynn McKeown

**Affiliations:** University of Leeds, Faculty of Medicine and Health, Leeds Institute of Cardiovascular and Metabolic Medicine, Leeds, LS2 9JT, UK

**Keywords:** Human *EFCAB4B* (Rab46 / CRACR2A), Mouse *Efcab4b*, Liver, Non-alcoholic steatohepatitis (NASH), Hepatocarcinoma (HCC), Non-alcoholic fatty liver disease (NAFLD)

## Abstract

*EFCAB4B* is an evolutionarily conserved protein that encodes for the Rab GTPase Rab46, and the CRAC channel modulator, CRACR2A. Previous genome wide association studies have demonstrated the association of *EFCAB4B* variants in the progression of non-alcoholic fatty liver disease (NAFLD). In this study we show that mice with global depletion of *Efcab4b*^*−/−*^ have significantly larger livers than their wild-type (WT) counterparts. We performed RNA-sequencing (RNA-seq) analysis of liver tissues to investigate differential global gene expression among *Efcab4b*^*−/−*^ and WT mice. Of the 69 differentially expressed genes (DEGs), analyses of biological processes found significant enrichment in liver and bile development, with 6 genes (*Pck1, Aacs, Onecut1, E2f8, Xbp1,* and *Hes1*) involved in both processes. Specific consideration of possible roles of DEGs or their products in NAFLD progression to (NASH) and hepatocarcinoma (HCC), demonstrated DEGs in the livers of *Efcab4b*^*−/−*^ mice had roles in molecular pathways including lipid metabolism, inflammation, ER stress and fibrosis. The results in this study provide additional insights into molecular mechanisms responsible for increasing susceptibility of liver injuries associated with *EFCAB4B*.

## Highlights

• *Efcab4b,* a gene with variations associated with non-alcoholic fatty liver disease, is successfully knocked out in mice.
• *Efcab4b* deletion in mice induces larger livers with inflammatory foci.
• *Efcab4b* deletion evokes differential changes in liver gene expression.
• Differentially expressed genes have roles in liver function and potential roles in pathological signaling pathways

## 1 Introduction

Non-alcoholic fatty liver disease (NAFLD), a chronic liver condition characterised by excessive fat in the liver, is associated with insulin resistance, obesity and lipidaemia [1]. In the UK, early-stage NAFLD may be present in 1 out of 3 people and is often asymptomatic. However, an increasing population of NAFLD patients will develop non-alcoholic steatohepatitis (NASH: defined as the presence of more than 5 % hepatic steatosis and inflammation with hepatocyte injury [2]). The persistence of inflammation can progress NASH to hepatic cirrhosis and hepatocellular carcinoma (HCC) [[3], [4], [5]]. Whilst NAFLD is the leading cause of liver fibrosis and HCC worldwide [6], it is also associated with the development of non-liver adverse outcomes such as cardiovascular diseases [[7], [8], [9]] and type 2 diabetes mellitus [10]. In addition to the effect on wellbeing and quality of life, the total economic costs of diagnosed NASH in the UK is estimated to be £2.3 to £4.2 billion [11].

The progression of NAFLD to NASH is multifactorial, however, the underlying mechanisms are not well understood. Whilst a population of patients develop NASH some, with the same co-morbidities and risk factors, just have a fatty liver. This variation in disease progression suggests genetic predispositions and, indeed, studies have shown that genetic factors play a role in NAFLD pathogenesis [12,13]. Although a polygenic disease, common genetic variants have been consistently associated with increased risk of steatosis, particularly small nucleotide polymorphisms (SNPs) in Patatin-like phospholipase domain-containing protein 3 (*PNPLA3*), Lysophospholipase-like 1 (*LYPLAL1*)*,* protein phosphatase 1 regulatory subunit 3B (*PPP1R3B*)*,* Neurocan (*NCAN*) and glucokinase regulator (*GCKR*) [[14], [15], [16]]. However, an accumulation of fat is not the only risk factor for promoting the transition from non-alcoholic fatty liver to NASH, a central component is persistent inflammation [4,5]. Identifying the triggers of inflammation remains a major issue in the field. Recently a pilot GWAS in patients with NAFLD looked at genetic variants significantly associated with hepatic histology. Here they identified rs887304 on chromosome 12 in EF-Hand calcium Binding Protein 4B (*EFCAB4B*) (also known as Ca^2+^ Release-Activated Channel Regulator 2 A; CRACR2A) to be associated with lobular inflammation [17]. To elucidate a genetic predisposition to the pathogenesis and progression of NASH, Grove et al. explored multiple risk-associated alleles present in monozygotic twins that both developed NASH cirrhosis and revealed both patients were heterozygous for six SNPs, including rs887304 in *EFCAB4B* [18]. Moreover, rs887304 was one of 19 SNPs shown to be significantly associated with NAFLD in a pilot study in an Indian population [19].

*EFCAB4B* encodes for two functional proteins: CRACR2A (CRACR2A-S, CRACR2A-201) [20] and Rab46 (CRACR2A-L, CRACR2A-203) [21]. 887304 is located at the 3’ UTR of CRACR2A but is intronic in the Rab46 coding region and could potentially regulate isoform expression (https://www.ensembl.org/Homo_sapiens/Gene/Variation_Gene/Table?db=core;g=ENSG00000130038;r=12:3606633-3764819). CRACR2A has been shown to be a regulator of store-operated calcium in T-cells [22] whilst Rab46 regulates the trafficking of unique granules in endothelial cells (Weibel-Palade bodies) [23] and differential secretion in mast cells [24]. In endothelial cells, Rab46 acts as a brake to prevent the secretion of pre-stored pro-inflammatory components in response to non-thrombotic stimuli [23]. The depletion of Rab46 in endothelial cells could therefore lead to the increased inflammation necessary for NAFLD disease progression, especially since SNPs in *EFCAB4B* [25] and the presence of NAFLD [26] have been associated with extreme inflammatory responses to COVID-19, with significantly increased serum levels of Weibel-Palade body cargo such as von Willebrand factor and Angiopoietin 2 [27]. However, whilst a patient with biallelic mutations in EFCAB4B, where neither Rab46 or CRACR2A is expressed, displayed immunodeficiency due to a loss of function in T-cells [28], CRACR2A expression in neutrophils promotes neutrophil migration in inflammation [29]. Thereby, considering the roles of both Rab46 and CRACR2A in inflammation it is important to understand their contribution to liver function where chronic inflammation is vital for the progression of NAFLD. Here we utilized an *Efcab4b* global knockout mouse to explore the impact of CRACR2A and Rab46 depletion on liver and to identify some potential pathways that contribute to inflammation/progression of NAFLD.

## 2 Results

### 2.1 Animal model validation

**Fig. 1:**
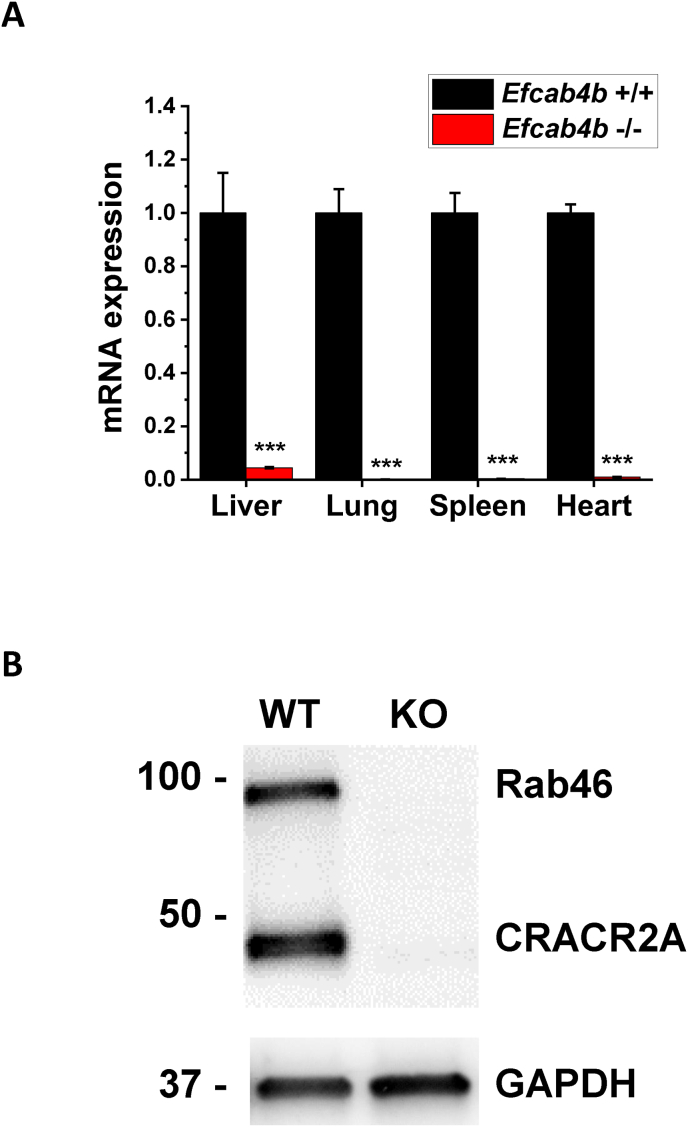
**Validation of *Efcab4b* gene knockout in mice.** (A) qPCR ΔCt analysis of *Efcab4b* expression relative to housekeeping genes and normalized to wild-type (WT) control in the liver, lungs, spleen and heart of *Efcab4b*^*−/−*^ mice and WT control mice. The abundance of mRNA encoding for both the CRACR2A and Rab46 protein isoforms is significantly decreased in all the tissues. n = 7 ∗∗∗p < 0.001. (B) Western Blot (full blot in Supplementary Fig. 1) of cell lysates from *Efcab4b*^+/+^ (WT) and *Efcab4b*^−/−^ (KO) mice depicting depletion of both Rab46 and CRACR2a proteins in KO mice. Loading control GAPDH.

**Fig. 2:**
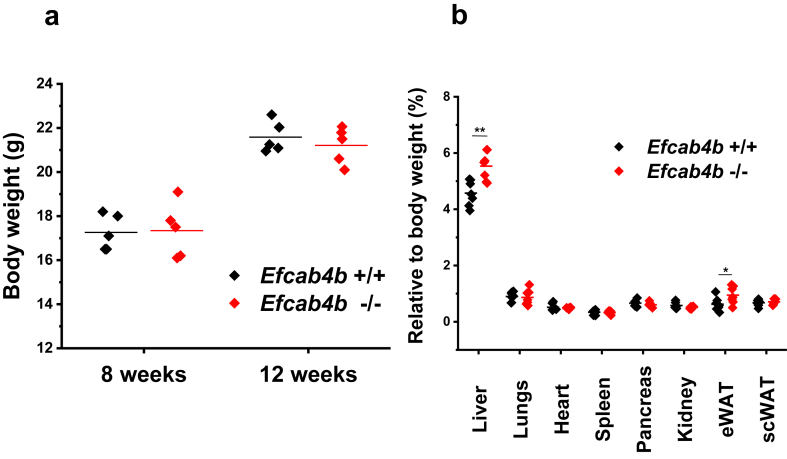
**Effect of *Efcab4b* gene knockout on body and organ weights.** a) Body weight at the age of 8 and 12 weeks of female control (WT: n = 5) and female *Efcab4b*^*−/−*^ mice (n = 5). Scatter plot shows body weight (g) of each mouse and straight line indicates the group mean. No significant difference is observed at these time points between WT (black diamonds) and knockout mice (red diamonds). b) Male and female mice were sacrificed at the age of 12 weeks and the main organs were harvested and weighted. Scatter plot shows data distribution of each organ from control and *Efcab4b*^*−/−*^ mice. Straight line indicates the mean value. Liver weight and eWAT weight is increased in *Efcab4b*^*−/−*^ mice compared to control (WT). n = 5 ∗∗p < 0,01 ∗p < 0,05 from Student t-test.

The *Efcab4b*^*−/−*^ mouse strain did not show any viability or fertility issues with regards to fertilisation and litter size. Mice with disrupted CRACR2A/Rab46 expression (*Efcab4b*^*−/−*^ mice) and respective WT controls were sacrificed at 12 weeks and gene knockout was validated from the stated tissues (Fig. 1a and b). Quantitative RT-PCR analysis of mRNA abundance in the liver, lung, spleen and heart from *Efcab4b*^*−/−*^ and WT mice showed reduced mRNA expression in all the stated organs, confirming global knockout of murine *Efcab4b* (Fig. 1a). Western Blotting demonstrated knockout of both Rab46 and CRACR2A isoforms at the protein level in cells from *Efcab4b*^+/+^ and *Efcab4b*^−/−^ mice (Fig. 1b, Supplementary Fig. 1A). Immunoblotting using an antibody that recognises both isoforms exhibited specific staining at the expected molecular weight (95 kDa) corresponding to Rab46 in control HUVECs and reduced intensity in *Efcab4b*^−/−^. *Efcab4b*^*−/−*^ mice appeared superficially normal in physical appearance compared to WT mice with no difference in life span up to 12 weeks between genotypes. Mice fed with standard chow diet had their body weight assessed at 8 and 12 weeks (Fig. 2a). No difference was seen in body weight at the considered time points between WT (*Efcab4b*^*+/+*^) and *Efcab4b*^*−/−*^ mice. A significant difference was observed in liver weight, where *Efcab4b*^*−/−*^ mice have increased liver mass compared to WT mice (Fig. 2b). A small but significant increase in the visceral fat was also observed in *Efcab4b*^*−/−*^ mice.

**Fig. 3:**
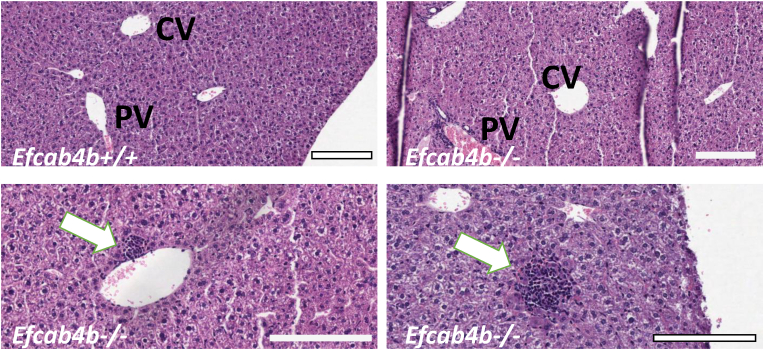
**Histological examination of liver of *Efcab4b* KO mice compared to control (WT).** Mice were sacrificed at the age of 12 weeks and the liver was harvested and weighed. Liver tissues were examined by H&E staining. Liver from WT *Efcab4b*^*+/+*^ control group revealing normal morphology with central vein (CV) and normal triad structure with portal vein (PV); Liver from *Efcab4b*^*−/−*^ mice reveals multiple focal inflammation sites with immune cell infiltration (white arrows) and portal vein and sinusoids congestion.

Histological analysis of livers from the WT mice indicated a normal liver lobular architecture with central vein and surrounding hepatocytes, sinusoids and nucleus (Fig. 3). *Efcab4b*^*−/−*^ mice show normal liver morphology, however hepatic mononuclear cell infiltration, congestion of portal vein and blood sinusoids appear to be a common feature. Accumulation of fat droplets was not detected in any of the analysed tissues section. The presence of focal periportal immune cell infiltration, with enlargement of the portal tract may suggest an early inflammatory condition due to gene deletion (Fig. 3).

### 2.2 Transcriptional changes suggest *Efcab4b* transcripts contribute to liver physiology

Having defined some of the morphological changes in the liver of *Efcab4b*^*−/−*^ mice we undertook bulk RNA-seq analysis of the livers extracted from WT control and *Efcab4b*^*−/−*^ mice. We compared the gene expression levels and associated functions of the genes differentially expressed between the two groups, to understand the influence of CRACR2A and Rab46 protein deficiency on mouse liver function.

**Fig. 4:**
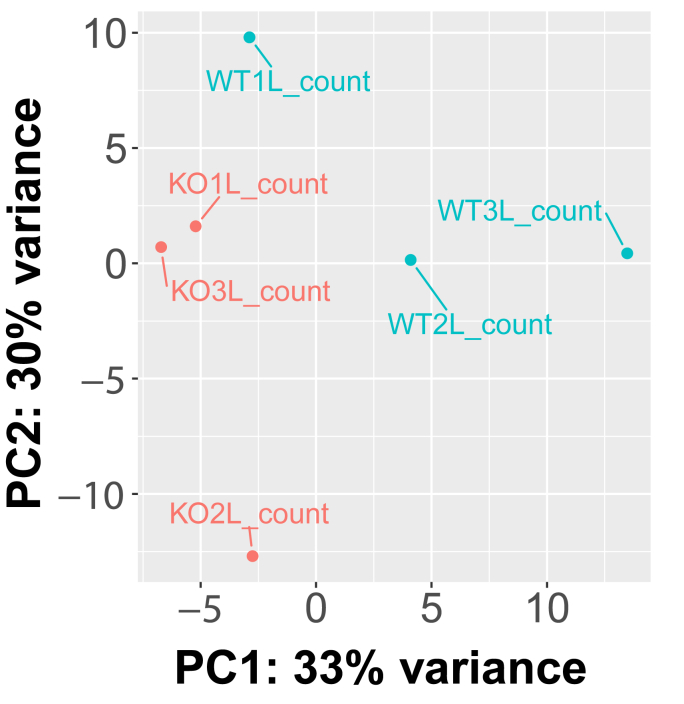
**PCA plot showing segregation of samples based on genome-wide expression profiles.** Each dot represents one sample, blue colour dots representing wild-type samples and red colour dots representing knockout samples.

A principal component analysis (PCA) demonstrated the source of greatest variation in transcriptional response of liver tissue (Fig. 4) was source of tissue The analysis revealed that PC1 accounted for 33 % of the variance, indicating a substantial contribution to the overall genetic variation between the samples. Similarly, PC2 explained 30 % of the variance, further capturing significant differences in the genetic profiles. By comparing three knockout samples to three control samples, distinct patterns and clustering were observed in the PCA plot, suggesting notable genetic distinctions between the two groups.

**Fig. 5:**
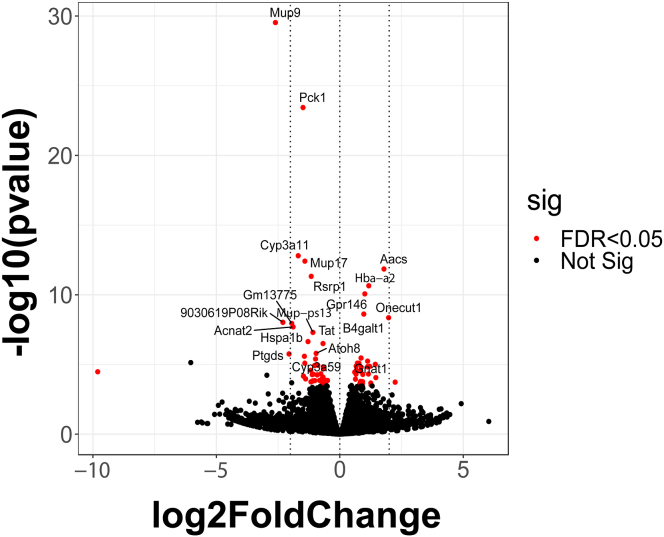
**Volcano plot showing differentially expressed genes** based on adjusted P-value<0.05. There are 69 differentially expressed genes (DEGs) detected comparing between knocked-out and wild-type. Of those 69 DEGs, 30 DEGs are upregulated and 39 DEGs are downregulated in knocked-out model.

**Fig. 6:**
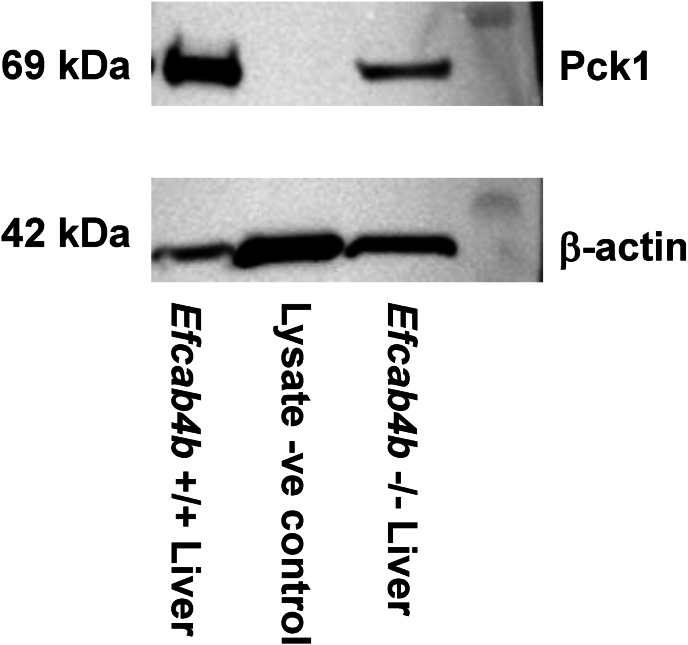
**Western Blot** (full blots shown in Supplementary Fig. 1) demonstrating decreased expression of Pck1 protein in liver tissue lysates from *Efcab4b*^−/−^ (KO) mice compared to *Efcab4b*^+/+^ (WT) mice. Negative cell lysate control and β-actin loading control.

Our study focused on comparing *Efcab4b*^*−/−*^ and WT models, revealing a total of 69 DEGs (Fig. 5). Among these DEGs, 30 were found to be upregulated while 39 were downregulated in the KO model. The full name of the top 30 differentially regulated genes in the *Efcab4b*^*−/−*^ livers compared to *Efcab4*^*+/+*^ (plus Log2 fold changes values and FDR) and their functional characteristics are shown in Table 2. An example of the differential expression of Pck1 at the protein level are demonstrated in Fig. 6 and Supplementary Fig. 1.

**Table 1:** PCR primers.

Primer target	Sequence
*m*-Cracr2a	F: 5′ CTGGAGCGACTCAATCAGAAGC 3′
	R: 5′ GAGGCAAGCTGAGTTGGAAGAG 3′
*m*-Rab46	F: 5′ GGGCAGCCTGTTGGAAAAGA 3′
	R: 5′ ACTCGGTAGTCGATGCCCAC 3′
*m*-Rab46 and Cracr2a	F: 5′ GATGGACAGACTTGGAGCCC 3′
	R: 5′ CAGCAATTTTCTTTCTGAGGGCA 3′

**Table 2:** The top 30 significantly differentially expressed genes (DEGs) based on adjusted p-value.

	Gene Symbol	log_2_FC	pADJ
1	Mup9	−2.6075	3.1900E-26
2	Pck1	−1.4881	1.9800E-20
3	Cyp3a11	−1.6837	5.7200E-10
4	Mup17	−1.4155	1.0400E-09
5	Aacs	1.7884	3.0600E-09
6	Rsrp1	−1.1571	8.5500E-09
7	Hba-a2	1.1723	3.4900E-08
8	Gpr146	1.0139	1.1700E-07
9	B4galt1	0.9729	2.9000E-06
10	Onecut1	1.9776	4.7300E-06
11	9030619P08Rik	−2.3037	9.3100E-06
12	Gm13775	−1.9459	1.0300E-05
13	Acnat2	−1.8897	1.6800E-05
14	Mup-ps13	−1.0885	3.8500E-05
15	Hspa1b	−1.2885	0.0002
16	Tat	−0.6794	0.0002
17	Atoh8	−0.9598	0.0010
18	Ptgds	−2.0571	0.0011
19	Cyp3a59	−1.4336	0.0015
20	Gnat1	0.8614	0.0019
21	Ces2c	−0.9825	0.0021
22	Loxl4	1.1242	0.0028
23	Serpina4-ps1	0.7744	0.0035
24	Mvk	0.7144	0.0035
25	Gm49395	−1.4180	0.0035
26	E2f8	1.4379	0.0040
27	Mup7	−0.8927	0.0045
28	Dio1	−1.0201	0.0046
29	Xbp1	0.6627	0.0049
30	Fgfr1	1.2086	0.0049

### 2.3 Bioinformatic pathway analyses

**Fig. 7:**
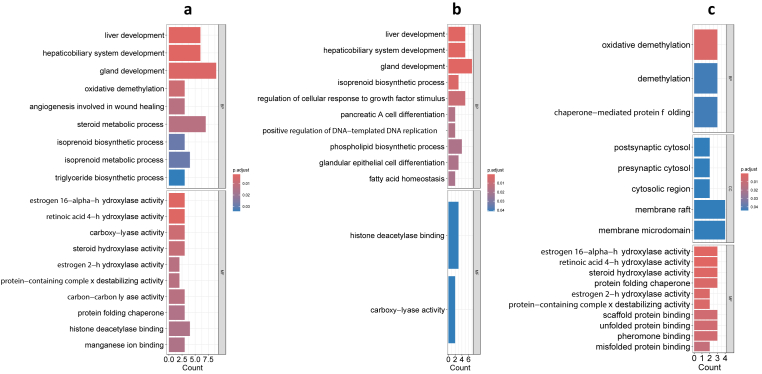
**Enrichment analysis using differentially expressed genes.** (a) Enrichment analysis of all DEGS; (b) enrichment analysis of upregulated DEGs; (c) enrichment analysis of downregulated DEGs. BP: biological processes, MF: molecular functions, and CC: cellular components.

Enrichment analysis was performed to gain insights into the functional significance of different Gene Ontology (GO) terms. Using all differentially expressed genes, we identified 55 significant terms based on adjusted P-value 0.05 (Fig. 7a). The analysis revealed several enriched biological processes (BP) and molecular functions (MF) associated with the studied genes. In terms of biological processes, the study found significant enrichment in liver development (GO:0001889) and hepaticobiliary system development (GO:0061008), with 6 common genes (*Pck1, Aacs, Onecut1, E2f8, Xbp1*, and *Hes1*) being involved in both processes. Gland development (GO:0048732) was also enriched, with 9 genes (*Pck1, Aacs*, *Onecut1, E2f8, Xbp1, Fgfr1, Hes1, Cd44*, and *Socs2*) associated with this process. Furthermore, the enrichment analysis highlighted molecular functions such as estrogen 16-alpha-hydroxylase activity (GO:0101020) and retinoic acid 4-hydroxylase activity (GO:0008401), both mediated by the genes *Cyp3a11, Cyp3a59*, and *Cyp3a25*. Other enriched molecular functions included protein folding chaperone activity (GO:0044183), histone deacetylase binding (GO:0042826), and magnesium ion binding (GO:0000287).

Using only the upregulated differentially expressed genes. The analysis revealed 36 significantly enriched GO terms related to various biological processes and molecular functions (Fig. 7b). In terms of biological processes, liver development (GO:0001889) and hepaticobiliary system development (GO:0061008) were found to be significantly enriched, Additionally, gland development (GO:0048732) showed enrichment. These findings suggest that the upregulated genes are associated with the development and function of liver and glandular tissues. Furthermore, the analysis revealed enrichment of genes involved in isoprenoid biosynthetic process (GO:0008299) and phospholipid biosynthetic process (GO:0008654). Genes related to monosaccharide metabolic process (GO:0005996) and hexose metabolic process (GO:0019318) were also enriched, indicating the involvement of upregulated genes in carbohydrate metabolism. Moreover, several enriched GO terms related to cellular signaling and regulation were identified. These included the regulation of cellular response to growth factor stimulus (GO:0090287) and the regulation of transforming growth factor beta receptor signaling pathway (GO:0017015). These findings suggest that the upregulated genes may play a role in modulating cellular responses to growth factors and signaling pathways. In terms of molecular functions, histone deacetylase binding (GO:0042826) and carboxy-lyase activity (GO:0016831) were significantly enriched. These findings indicate that the upregulated genes may be involved in epigenetic regulation and catalytic activities.

The enrichment analysis of the downregulated genes revealed 24 significant GO terms (Fig. 7c). In terms of biological processes, the downregulated genes were enriched in processes such as oxidative demethylation (GO:0070989) and demethylation (GO:0070988), suggesting potential involvement in the regulation of methylation processes. Additionally, the downregulated genes were associated with chaperone-mediated protein folding (GO:0061077), indicating a disruption in protein folding mechanisms. In terms of cellular components, the downregulated genes were enriched in regions such as postsynaptic cytosol (GO:0099524), presynaptic cytosol (GO:0099523), cytosolic region (GO:0099522), membrane raft (GO:0045121), and membrane microdomain (GO:0098857), suggesting potential alterations in synaptic and membrane organization. The molecular function analysis revealed enrichment of activities such as estrogen 16-alpha-hydroxylase activity (GO:0101020), retinoic acid 4-hydroxylase activity (GO:0008401), steroid hydroxylase activity (GO:0008395), protein folding chaperone (GO:0044183), and scaffold protein binding (GO:0097110).

**Fig. 8:**
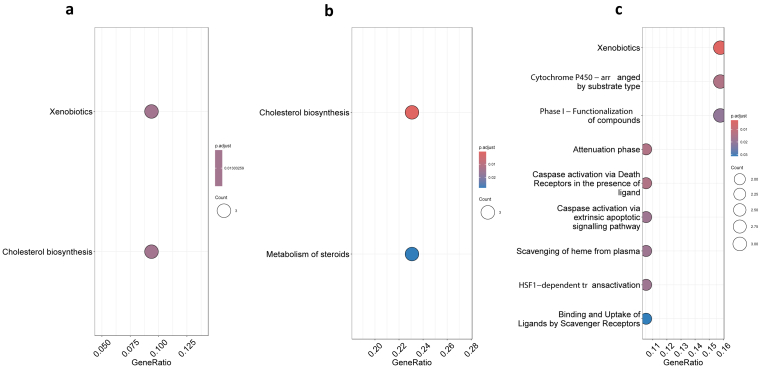
**REACTOME analysis of all differentially expressed genes.** (a) REACTOME analysis of all DEGS; (b) REACTOME analysis of upregulated DEGs; (c) REACTOME analysis of downregulated DEGs. The size of the circle represents the number of genes enriched in the pathway and the colour of the circle indicates the level of significance (P < 0.05).

We performed a pathway enrichment analysis of all differentially expressed genes using the REACTOME database (Fig. 8a). The analysis revealed enrichment of genes involved in Xenobiotics (R-MMU-211981) metabolism, with the downregulated genes including *Cyp3a11, Cyp3a59,* and *Cyp3a25*. Additionally, the cholesterol biosynthesis (R-MMU-191273) pathway showed enrichment, with *Mvk, Pmvk,* and *Idi1*.

REACTOME pathway analysis was performed using upregulated genes (Fig. 8b). This analysis revealed two significant pathways were enriched by the differentially expressed genes. The first pathway, Cholesterol biosynthesis (R-MMU-191273), involves the synthesis of cholesterol, a crucial lipid molecule with diverse cellular functions. Out of the 28 upregulated genes tested, three genes (*Mvk, Pmvk,* and *Idi1*) were found to be associated with this pathway. The second significant pathway, the Metabolism of steroids (R-MMU-8957322), involves various processes related to the metabolism of steroid molecules. Similarly, three genes (*Mvk, Pmvk*, and *Idi1*) were identified as associated with this pathway.

The REACTOME analysis using downregulated genes identified significantly enriched pathways (Fig. 8c). Among them, the Xenobiotics pathway (R-MMU-211981) showed enrichment, indicating the potential involvement of *Cyp3a11, Cyp3a59,* and *Cyp3a25* genes. Additionally, the Attenuation phase pathway (R-MMU-3371568) exhibited enrichment, with *Hspa1b* and *Hspa8* genes among the upregulated genes. Furthermore, the pathway Cytochrome P450 - arranged by substrate type (R-MMU-211897) demonstrated enrichment, suggesting potential dysregulation of *Cyp3a11, Cyp3a59,* and *Cyp3a25*. Pathways associated with apoptosis, such as Caspase activation via Death Receptors in the presence of ligand (R-MMU-140534) and Caspase activation via extrinsic apoptotic signaling pathway (R-MMU-5357769), were also enriched, with *Ly96* and *Casp8* among the upregulated genes. Additionally, the pathway Scavenging of heme from plasma (R-MMU-2168880) and Binding and Uptake of Ligands by Scavenger Receptors (R-MMU-2173782) showed enrichment, suggesting potential dysregulation of *Cd163, Apol9a*, and *Hspa1b/Hspa8*, respectively.

## 3 Discussion

Variants in *EFCAB4B* gene have been implicated in the development of NAFLD and variants in the proteins CRACR2A and Rab46 play roles in inflammation and in diseases enhanced by inflammation. Since we observed that mice lacking the *Efcab4b* gene appeared phenotypically normal except the size and weight of the livers and the epididymal fat pads, we hypothesized that *Efcab4b* deficiency could impact the expression of genes important for liver development and function. In this study, we examined the hepatic transcriptome of *Efcab4b* deficient mice and identified biological functions and genes associated with hepatotoxicity, lipid metabolism, and metabolic disorders. In particularly, we demonstrated roles for DEGS in liver and bile development which could give an insight in to the large livers observed in the *Efcab4b*^*−/−*^ *mice.*

### 3.1 Liver disease related genes

**Fig. 9:**
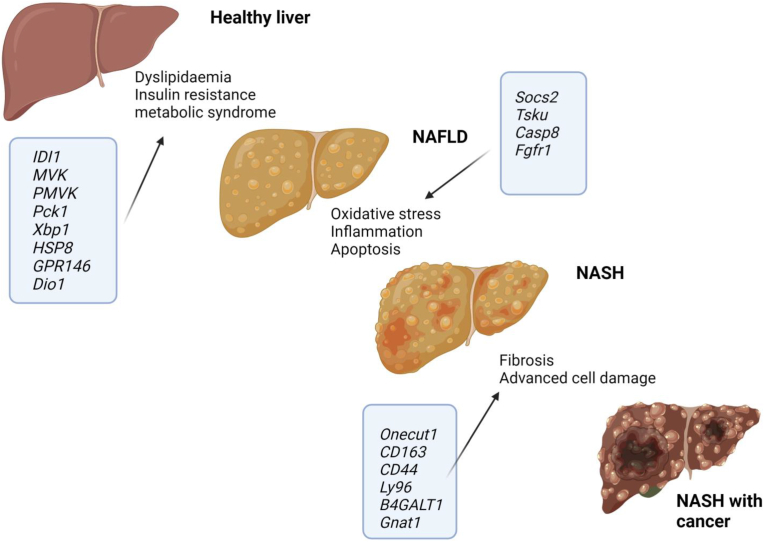
**Schematic depicting the differentially expressed genes associated with molecular pathways that evoke the progression of NAFLD to NASH and HCC.** Created with Biorender.com. NAFLD = non-alcoholic fatty liver disease. NASH = non-alcoholic steatohepatitis. HCC = Hepatocellular carcinoma.

Whilst our data is preliminary to in vivo studies, we explored if the DEGs we observed in *Efcab4b*^*−/−*^ liver tissue had translational products that had previously been reported to play a role in the progression of liver disease. We considered the molecular pathways that promote the progression of a healthy liver to a cirrhotic liver, and which pathways DEGs have been previously shown to have a role in (Fig. 9). A simple NAFLD/NASH-based literature search revealed associations between 32 DEGs and NAFLD/NASH/HCC (Table 3, Table 4) and the potential role they play in NAFLD progression (Fig. 9).

**Table 3:** Upregulated genes with roles in pathways associated with NAFLD progression.

GENE SYMBOL	LOG_2_FC	PADJ	LIVER DISEASE ROLE	GENE DESCRIPTION	EXPRESSION IN LIVER DISEASE	REF
Socs2	2.24	0.0323	NASH	Suppressor of cytokine signaling 2	Down	[[30], [31], [32]]
Onecut1	1.98	0	HCC	One cut domain, family member 1	Down	[33]
E2f8	1.44	0.004	HCC	E2F transcription factor 8	Up	[34]
Fgfr1	1.21	0.0049	Steatosis/HCC	Fibroblast growth factor receptor 1	Up	[35,36]
Hes1	1.16	0.0123	HCC	Hes family bHLH transcription factor 1	Up	[37]
Sik1	1.13	0.0049	steatosis	Salt inducible kinase 1	Down	[38]
Loxl4	1.12	0.0028	HCC	Lysyl oxidase-like 4	Up	[39]
Gpr146	1.01	0	Steatosis	G protein-coupled receptor 146	Down	[40]
B4galt1	0.97	0	HCC	UDP-Gal:betaGlcNAc beta 1,4- galactosyltransferase, polypeptide 1	Down	[41]
Vgll4	0.92	0.0311	HCC	Vestigial like family member 4	Up	[42]
Idi1	0.92	0.0374	Steatosis	Isopentenyl-diphosphate delta isomerase	Up	[43]
Prss8	0.87	0.005	Steatosis	Protease, serine 8 (prostasin)	Down	[44]
Gnat1	0.86	0.0019	HCC	Guanine nucleotide binding protein, alpha transducing 1	Down	[45]
Odc1	0.84	0.03	HCC	Ornithine decarboxylase, structural 1	Up	[46]
Pmvk	0.67	0.0076	Steatosis	Phosphomevalonate kinase	Up	[47]
Xbp1	0.66	0.0049	Steatosis	X-box binding protein 1	Up	[48,49]

NASH: non-alcoholic steatohepatitis; HCC: Hepatocarcinoma.

**Table 4:** Downregulated genes with roles in pathways associated with NAFLD progression.

GENE SYMBOL	LOG_2_FC	PADJ	LIVER DISEASE ROLE	GENE DESCRIPTION	EXPRESSION IN LIVER DISEASE	REF
Acnat2	−1.89	2.00E-05	Steatosis	Acyl-coenzyme A amino acid N-acyltransferase 2	Up	[50]
Cyp3a11	−1.68	0.00 E+00	Steatosis	Cytochrome P450, family 3, subfamily a, polypeptide 11	Up	[51]
Pck1	−1.49	0.00 E+00	Steatosis	Phosphoenolpyruvate carboxykinase 1, cytosolic	Down	[52,53]
Igfbp6	−1.39	0.0223	Steatosis	Insulin-like growth factor binding protein 6	Up	[54]
Hspa1b	−1.29	0.0002	NASH HCC	Heat shock protein 1B	Up	[55]
Cd163	−1.18	0.0076	HCC	CD163 antigen	Up	[56,57]
Ly96	−1.17	0.0313	NASH HCC	Lymphocyte antigen 96	Up	[58,59]
Cd44	−1.1	0.0283	HCC	CD44 antigen	Up	[60,61]
Tsku	−1.07	0.0124	NASH	Tsukushi, small leucine rich proteoglycan	Up	[62]
Dio1	−1.02	0.0046	Steatosis	Deiodinase, iodothyronine, type I	Up	[63]
Ces2c	−0.98	0.0021	Steatosis	Carboxylesterase 2C	Down	[64]
Atoh8	−0.96	0.0010	HCC	Atonal bHLH transcription factor 8	Down	[65]
Casp8	−0.72	0.0359	NASH	Caspase 8	Up	[66,67]
Baiap2	−0.71	0.0218	HCC	Brain-specific angiogenesis inhibitor 1-associated protein 2	Up	[68]
Slc25a47	−0.68	0.0178	HCC	Solute carrier family 25, member 47	Down	[69]
Hspa8	−0.67	0.0049	HCC	Heat shock protein 8	Up	[55]

NASH: non-alcoholic steatohepatitis; HCC: Hepatocarcinoma.

***Lipid metabolism and steatosis***: Several identified DEGs suggest an association between *Efcab4b*^*−/−*^ and the first stage of NAFLD: that is simple steatosis where the accumulation of lipid droplets within hepatocytes is associated with negligible, if any, inflammation. Several DEGs play roles in lipid metabolism and therefore have the potential to promote dyslipidaemias. For example, expression of inhibitor of DNA binding1 (*ID1*), phosphomevalonate kinase (*PMVK*), G protein-coupled receptor 146 (*GPR146*) [40] and X-box binding protein-1 (*XBP1*) are upregulated and phosphoenolpyruvate carboxykinase 1 (*PCK1*) downregulated in NAFLD or lipid disorders, this is reflected in the livers of *Efcab4b*^*−/−*^ mice as compared to WT. *Efcab4b* depletion induced 1.488x downregulation in the expression of *Pck1*. *PCK1* is a gene that plays a critical role in hepatic glucose metabolism. It is involved in gluconeogenesis, the process by which the liver produces glucose from non-carbohydrate precursors, by catalyzing the conversion of oxaloacetate to phosphoenolpyruvate, a key step in glucose synthesis. Murine depletion of *Pck1* promotes metabolic-associated fatty liver disease (MAFLD) [52] and *PCK1* is down regulated in hepatocytes extracted from human diseased liver [53]. Moreover, dietary plant sterols (phytosterols), that have cholesterol-lowering properties and attenuate deleterious effects of cholesterol overload, can rescue the impact of an HFD in a hamster model by restoring hepatic *Pck1* expression [70]. *ID1* and *PMVK* genes encode for enzymes that are crucial for the cholesterol biosynthesis pathway and these genes are upregulated in the *Efcab4b*^*−/−*^ mice versus WT mice (Table 3). Hepatic free cholesterol overload results in cholesterol-associated steatohepatitis and may play an important role in the development and progression of NAFLD, NASH and hepatic cancer [71]. Statins appear to provide significant benefit in preventing progression to NASH and NASH-cirrhosis, suggesting in addition to cholesterol in the diet, the biosynthesis pathway plays a role. Betaine, a drug known to effectively improve hepatic lipid metabolism, evokes depletion of *IDI1* [43], whilst gypenosides, natural drugs used to treat lipid disorders, reduce the expression of *PMVK* [47]. The upregulation of the transcription factor *Xbp1*, in the *Efcab4b*^*−/−*^ mice has also been demonstrated in samples from patients with NAFLD, NASH and HCC [48]. Hepatocyte-specific *Xbp1* deficiency inhibited the development of steatohepatitis in mice fed the high-fat diet whilst macrophage-specific *Xbp1* knockout mice developed less severe steatohepatitis and fibrosis than wild-type *Xbp1* mice. *XBP1* is the key transcription factor for initiating the unfolded protein response (UPR) in response to ER stress. In addition to being required for de novo fatty acid synthesis in the liver (a function unrelated to its role in the UPR [49]), upon ER stress *XBP1* specifically induces expression of the transcription factor *FOXA3* and exacerbates lipid accumulation, linking ER stress to NAFLD progression [72]. The analogous changes of these genes in *Efcab4b*^*−/−*^ mice suggest the depletion of *Efcab4b* could evoke NAFLD progression by impacting lipid homeostasis. However, some DEGs that are positively associated with hepatic steatosis such as deiodinase 1 (*Dio1*) [63], are downregulated in the *Efcab4b*^*−/−*^ livers, in addition the histological staining of the livers from *Efcab4b*^*−/−*^ or WT mice did not show any pronounced lipid accumulation (in this particular study). Thereby we questioned if the identified DEGs could play a role in the progression of NAFLD from a simple macrovesicular steatosis to NASH and eventually hepatocarcinoma (HCC).

***Inflammation and ER stress in NASH*:** Progression from NAFLD to NASH involves other damaging factors, such as activation of inflammatory pathways, ER stress and dysregulated hepatocyte apoptosis. Several of the DEGs identified in the liver of *Efcab4b*^*−/−*^ mice could play roles in these processes, however in these instances the expression in *Efcab4b*^*−/−*^ liver contrasts that of disease. For example, in NASH, suppressor of cytokine signaling 2 (*SOCS2*) is down regulated whilst Tsukushi (*TSKU*), and Caspase 8 (*CASP8)* are upregulated. The contrasting expression levels of these genes in the livers of *Efcab4b*^*−/−*^ mice suggest that the deletion of *Efcab4b* could be protective against inflammation. *Socs2* displays the highest fold upregulation (2.24x) in *Efcab4b*^*−/−*^ mice. *SOCS2* is one of classic negative regulators of cytokine signaling, which has recently been described as an anti-inflammatory mediator. In human samples the level of *SOCS2* expression was negatively correlated with NASH: *SOCS2* overexpression in macrophages suppressed inflammation and apoptosis via inhibiting NF-κB signaling pathway, whilst *SOCS2* knock-down in macrophages caused an increased activation of NF-κB. In addition, *SOCS2* expression in macrophages also suppressed inflammation via limiting the activation of inflammasomes, strongly indicating that *SOCS2* plays a role in inhibiting inflammation and apoptosis via NF-κB and inflammasome signaling pathway in macrophages during NASH [30]. Similarly, in HCC samples, immunohistochemical staining demonstrated lower levels of *SOCS2* protein expression in patients with HCC [31], whilst Liu et al. suggested *SOCS2* is a protective factor, because its high expression improves the prognosis of HCC patients [32]. Caspase 8 (*CASP8*) is essential for death-receptor-mediated apoptosis activity apoptosis, a critical mechanism contributing to inflammation and fibrogenesis, therefore its modulation is critical for the pathogenesis of NASH. Surprisingly the *Efcab4b*^*−/−*^ mouse showed a 0.71x downregulation of *Casp8* expression which is protective in the development of NASH in *Casp8* knockout mice. Here, the lack of *Casp8* expression in hepatocytes reduced the diet-dependent increase in apoptosis and decreased expression of proinflammatory cytokines as well as hepatic infiltration. As a consequence, ROS production was lower, leading to a reduction in the progression of liver fibrosis in *Casp8*^*−/−*^ livers [66]. In agreement, curcumin treatment was shown to be beneficial in preventing the development of NASH in rat models by reducing apoptosis and decreasing the expression of *Casp8* [73]. *TSK* is a hepatokine induced in response to both endoplasmic reticulum stress and inflammation in severely obese mice. In humans, hepatic TSK expression and increased serum levels are also associated with steatosis and acute liver failure [62]. TSK level is downregulated by 1.07x in the livers from *Efcab4b*^*−/−*^ mice versus WT mice.

***Fibrosis and HCC***: Our RNAseq analysis of the livers from *Efcab4b*^*−/−*^ mice exhibit downregulation of the macrophage marker CD163, the CD44 antigen and Lymphocyte antigen 96 (*Ly96*) and upregulation of beta-1, 4-galactosyltransferase 1 (*B4GALT1*). In previous studies increased circulating levels of CD163 in patients with HCC and in diabetic patients with advanced NASH fibrosis, suggests CD163 as a biomarker for disease severity in NAFLD [74,56]. CD44 is a cell surface antigen that acts as a co-receptor in tyrosine kinase receptor signaling and is thereby involved in cell-cell interactions cell adhesion and migration. It is highly expressed in cancer cells and promotes tumour progression. Accordingly the upregulation of CD44 is observed in HFD diet fed animals displaying inflammation, fibrosis and is particularly significantly associated with the malignant transformation of hepatocytes in NAFLD [75,76]. The deletion of *CD44* inhibits metastasis formation in mice [60] and in obese patients, hepatic CD44 and serum sCD44 strongly correlated with NASH [77]. Indeed, CD44 is considered a cancer stem cell marker [78] where immunohistochemical analysis of human HCC samples demonstrate increased expression of CD44. Ly96 (also known as myeloid differentiation factor 2: MD2) is a co-receptor for Toll-like receptor 4 (TLR4). Together they are key in recognition of lipopolysaccharide (LPS) and activation of proinflammatory pathways. In a mouse model of NASH, knockout of *Ly96* significantly attenuated triglyceride accumulation, lipid peroxidation, inflammation and liver fibrosis [58]. Similarly an angiotensin II liver injury mouse model displayed significantly reduced inflammation and fibrosis [59]. *B4GALT1* is significantly down regulated in the livers of patients with HCC and reducing *B4GALT1* enhanced HCC cell migration and invasion in vitro and promoted lung metastasis of HCC in NOD/SCID mice [41].

Comparing the level of gene expression (i.e. those that are upregulated in disease (yellow) or down regulated in disease (blue)) between previous NAFLD/NASH/HCC studies (Table 3, Table 4), to the expression levels in the livers of the *Efcab4b*^*−/−*^ mouse, demonstrates that of the 33 genes, 14 genes are expressed in a similar manner in the *Efcab4b*^*−/−*^ mice, whilst 19 are contradictory. Particularly, DEGs that have been shown to have roles in molecular pathways leading to NASH, through inflammation, are expressed in a contradictory manner suggesting depletion of *Efcab4b* could be protective especially as previous studies suggest a role for Rab46 in inflammation. However, although we have shown that changes in Pck1 protein expression reflects the reduction in gene expression, an integrated proteomics approach would link gene expression changes with functional outcomes and could explain possible discrepancies between gene expression and disease trajectory. Additionally, depletion of the *Efcab4b* gene prevents expression of both CRACR2A and Rab46 protein isoforms which may differentially affect both gene expression and liver function. Thereby, our future studies will employ CRISPr technology in cells to determine the effect of the NAFLD-associated SNP rs887304 (which is located on a possible splicing site where these two proteins diverse) on isoform expression. We will expand this technology to develop mouse models to gain insight the specific roles of these two proteins on liver disease by using diet induced NAFLD. Moreover, the development of liver-specific conditional knockout and inducible expression systems will overcome some of the limitations of using a global knockout where the effect of *Efcab4b* depletion could be indirect.

Despite numerous studies associating *EFCAB4B* with NAFLD, the underlying molecular mechanisms are unknown. This study was undertaken to highlight genes and therefore molecular pathways that have the potential to play a role in *EFCAB4B* associated progression of liver disease. Future studies will include measurements of serum transaminases and other broader tests like bilirubin, to clarify the impact of *Efcab4b* depletion on liver health. Moreover, because our histological data suggests the presence of inflammation, measurement of inflammatory markers such as C-reactive protein, TNF-α and interleukins (IL6, IL-1β) along with further histological analysis of lipids and specific staining of inflammatory cell types will be undertaken to confirm an inflammatory response. To specifically define the underlying molecular pathways, we will use this data and, integrated with our proteomic studies, will undertake a targeted approach, using the identified DEGs as targets for CRISPR-/CAS9 or RNAi based functional screens and further validate their role in liver disease by investigating how these knockouts respond to high fat diets. These studies along with manipulation of Rab46/CRACR2A expression would lead to a greater understanding of the mechanisms by which *Efcab4b* impacts liver function in health and disease.

## 4 Conclusion

In summary, our results indicate that deletion of *Efcab4b* alters the transcriptional profile of the liver. The biological processes and signaling pathways associated with the differentially expressed transcripts found following *Efcab4b* depletion were related to certain molecular pathways involved in liver health and disease. Specifically, genes and processes related to liver and gland development, cholesterol biosynthesis and metabolism which may ultimately contribute to liver disease development. The identification of DEGs lays the ground for future studies to uncover the intricate regulatory mechanisms underlying the CRACR2A/Rab46 phenotype by using gene targeting approaches and integrating other omic-based analyses. The research undertaken here will lead to studies to understand the role of *Efcab4b* in the immune response and how it contributes to NAFLD progression. In addition, the generation of appropriate disease models in these mice will further support studies into the role of *Efcab4b* function in health and disease. Our findings may contribute to a better understanding of the molecular landscape associated with Rab46, opening avenues for further investigation into the biological processes and potential therapeutic targets associated with non-alcoholic fatty liver disease.

## 5 Methods

### 5.1 Study design

#### 5.1.1 Mice

Murine work was carried out in accordance with The Animals (Scientific Procedures) Act 1986 (Amended 2012). Mice were kept in the University of Leeds animal facility under standard conditions; housed in ventilated cages (GM500, Techniplast, West Chester, PA, USA, five mice per cage) with a 12 h light/dark cycle, 50–70 % humidity, and a temperature of 21 °C. They received a standard chow diet (CRM, Special Diet Services, Augy, France) and had access to water through Hydropac®, London, UK, pouches. Experiments were conducted under Home Office Project License P606230FB. All studies were approved by the University of Leeds Ethics Committee. Male and female animals were ear notched at weaning, with these samples subsequently used for genotyping.

No experimental procedures were performed on live mice and euthanasia was performed in accordance with ARRIVE guidelines.

#### 5.1.2 Generation of *Efcab4b*^*−/−*^ mice

The *Efcab4b* knockout mouse strain was created from ES cell clone 15424 A-C4, generated by Regeneron Pharmaceuticals, Inc. and litters obtained from the KOMP Repository (www.komp.org). The homozygous C57BL/6N-CRACR2At^m1.1(KOMP)Vlcg^ and WT C57BL/6 N strains were achieved by heterozygous matings. Mice were depleted of the gene *Efcab4b* which prevents the expression of both CRACR2A and Rab46 protein. Genotyping was performed and homozygous and WT mice were selected for breeding. Mouse genotyping was performed by taking ear notches at weaning age. The samples were sent for automated genotype PCR service (Transnetyx, Cordova, TN, USA; see primer Table 1).

#### 5.1.3 Sampling

For histology, 6x homozygous KO (*Efcab4b*^−/−)^ and 7x WT (*Efcab4b*^+/+^) mice were weighed at 8 weeks and 12 weeks after which they were schedule 1 and organs were collected. The dissected organs were weighed prior to being snap frozen in liquid nitrogen and stored at in paraformaldehyde for histological examination. Organ-to-body weight ratios were calculated normalising the weight of each organ with its respective body weight.

#### 5.1.4 Histology

Liver samples were fixed in paraformaldehyde overnight and then kept in 70 % ethanol until further analysis. Liver tissues from female *Efcab4b*^*−/−*^ and WT mice were processed at the St. James's hospital histology facility (Leeds) who were blinded to the labelling. Histological examination of liver tissue was performed from the right lobe of the liver using Haematoxylin and Eosin (H&E) staining to reveal the main hepatic features. H&E staining was performed by the histology department at St. James's hospital and imaged slices were sent and opened with Aperio ImageScope for analysis. Identification of foci as areas of increased cellular staining was performed blind.

#### 5.1.5 Western Blotting

For quantitative experiments, the protein concentration in cell and liver tissue lysates were measured using a Bio-Rad Assay (Bio-Rad Laboratories) as per the manufacturer's protocol and by comparison against a BSA protein standard curve. Equal amounts (10–20 μg) of samples were mixed with a 4x sample loading buffer (200 mM Tris pH 6.8, 8 % SDS, 40 % glycerol, 8 % mercaptoethanol, 0.1 % bromophenol blue) and boiled for 5 min at 95 °C to fully denature the proteins. The protein samples were loaded alongside with a molecular weight ladder (Bio-Rad PrecisionPlus) on a 4–20 % gradient gel. Samples were resolved by SDS-PAGE at 120 V 1 h and transferred onto PVDF membranes (Millipore) by using wet transfer system for 50 min at 100 V. The membrane incubated in 5 % non-fat milk in TBS-T (145 mM NaCl, 20 mM Tris-base, 0.5 % Tween 20, pH 7.5) for 1 h to block non-specific binding sites. Subsequently, membranes were incubated overnight at 4 °C with primary antibodies (rabbit anti-CRACR2a: Proteintech 1:800; rabbit anti-Pck1: Fisher Scientific 16754-1-AP 1:10000). After 3x washes in TBST-T membranes were incubated with horse radish-peroxidase (HRP) donkey anti-mouse or rabbit secondary antibodies (1:5000) (Jackson ImmunoResearch) for 1 h at room temperature in 5 % milk. Membranes washed 4 times with TBS-T before visualization with SuperSignal Femto Detection Reagents (ThermoScientific) using GeneSys software. Membranes were re-probed for loading controls after stripping with buffer (RestoreTM- Stripping Buffer) for 20 min and the protocol repeated from the blocking stage (anti-β-actin 1:10,000: anti-GAPDH 1:10,000).

#### 5.1.6 RNA isolation

Liver tissue from 3x *Efcab4b*^*−/−*^ male mice 11 weeks and 3x *Efcab4b*^*+/+*^ male mice 11 weeks were placed in an RNA/DNAse free tube with a metal lysis bead with 1 ml Trizol Reagent. Tubes placed in a tissue lyser were agitated at 26 Hz for 3 × 1 min. The resultant liquid was transferred to 1.5 ml centrifuge tubes, 200 μl of phenol-chloroform was added for 3 min at room temperature, then spun at 12000g (4 °C) for 15 min. The supernatant (top aqueous phase) was transferred to new tubes and 500 μl of isopropanol added to each tube and incubated for 10 min. The samples were spun for 15 min at 12000g (4 °C), and the supernatant discarded. The pellets were re-suspended in 1 ml of 75 % ethanol (in dH2O) and centrifuged at 8000 g (4 °C) for 5 min. The supernatant was removed carefully, the pellet dried and then 20–50 μl of RNA-free dH2O was added to each sample and stored at −80 °C. RNA was quantified using a NanoDrop®.

#### 5.1.7 RNA sequencing and bioinformatics analysis

RNA extracted from the three samples of each of the two conditions; WT and *Efcab4b*^−/−^, were sequenced by Novogene, Cambridge, United Kingdom. The TruSeq Stranded mRNA kit (Illumina) was used for library preparation, following the manufacturer's recommended protocol. The libraries were sequenced on the HiSeq 4000 platform with 150 bp paired-end strategy. Original image data file was transformed to sequenced reads (Raw Data) and adaptor sequences and poly A tails were removed, generating raw reads in FASTQ format by Illumina CASAVA 1.8 base recognition. On average, 84.59 M high-quality reads were generated from the RNA sequencing project. The 24 raw reads were uploaded to the ENA-EMBL-EBI database under the accession number E-MTAB-13110.

STAR aligner [79] was used to map the raw reads to the *mouse* reference genome (Ensembl GRCm39) [80,81]. Since we had 3x biological replicates for each condition, differential gene expression levels were quantified by featureCounts [79,82] and normalized by DESeq2 [83] using the negative binomial model, this created false discovery rate (FDR) values of p = < 0.05 and log2 [foldchange] threshold >1. For pathway enrichment analyses, Kyoto Encyclopedia of Genes and Genomes (KEGG) and REACTOME databases were searched via clusterProfiler and ReactomePA, respectively, to predict potential enriched pathways. The significant terms were selected based on adjusted p-value (FDR) < 0.05. The principal component analysis plot, volcano plot and dot plots were generated using the GGPlot2 function in R.

## CRediT authorship contribution statement

**Chew W. Cheng:** Writing – review & editing, Writing – original draft, Visualization, Software, Methodology, Formal analysis. **Lucia Pedicini:** Writing – review & editing, Visualization, Methodology, Investigation, Formal analysis. **Cintli Morales Alcala:** Writing – review & editing, Resources. **Fenia Deligianni:** Writing – review & editing, Resources. **Jessica Smith:** Writing – review & editing, Resources. **Ryan D. Murray:** Investigation, Writing – review & editing. **Harriet J. Todd:** Formal analysis, Investigation. **Niamh Forde:** Writing – review & editing, Writing – original draft, Resources, Formal analysis. **Lynn McKeown:** Writing – original draft, Visualization, Supervision, Resources, Project administration, Methodology, Investigation, Funding acquisition, Formal analysis, Data curation, Conceptualization.

## Data availability

Via ArrayExpress on https://www.ebi.ac.uk/biostudies/arrayexpress/studies/E-MTAB-13110.

## Funding

The work was supported by a Medical Research Council grant to LM (MR/T004134/1). CWC is supported BHF Mautner Career Development fellowship.

## Declaration of competing interest

The authors declare that they have no known competing financial interests or personal relationships that could have appeared to influence the work reported in this paper.

## Appendix A Supplementary data

Fig. S1. (A) Full western blot using anti-CRACR2A antibody to detect expression of both CRACR2A and Rab46 in cell lysates extracted from *Efcab4b*^+/+^ (BMMCs WT) and *Efcab4*^−/−^ (BMMCs KO) mice. HUVECs used as a positive control for Rab46 staining and LAD2 cells as a negative control. The bands for detection in the mouse cell lysates were enhanced to visualize (arrow) since GAPDH (loading control) demonstrated less protein in these as compared to the control groups. (B) Full western blots of Pck1 and β-actin as a loading control in liver tissue lysates from *Efcab4b*^+/+^ (WT) and *Efcab4b*^−/−^ (KO). Fig. S1. (A)
