# Supplementary figures and images for: RNA-seq analysis reveals transcriptome changes in livers from Efcab4b knockout mice
Source: Biochem Biophys Rep. 2025 Feb 15;41:101944. doi: 10.1016/j.bbrep.2025.101944 (PMC11872658; doi:10.1016/j.bbrep.2025.101944)

## Slide 1
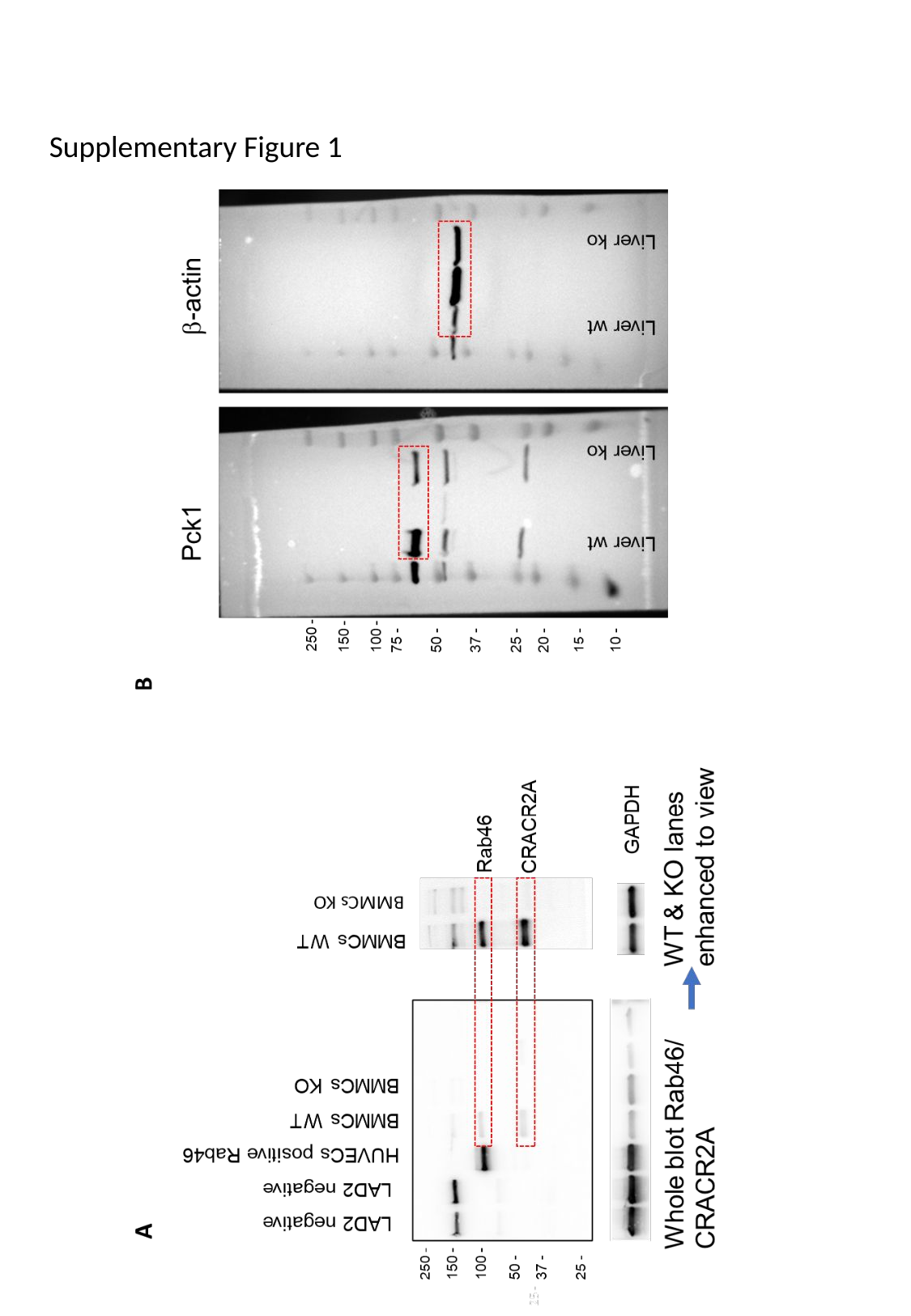

Supplementary Figure 1

Supplement: Fig. S1. (A) — Full western blot using anti-CRACR2A antibody to detect expression of both CRACR2A and Rab46 in cell lysates extracted from Efcab4b+/+ (BMMCs WT) and Efcab4−/− (BMMCs KO) mice. HUVECs used as a positive control for Rab46 staining and LAD2 cells as a negative control. The bands for detection in the mouse cell lysates were enhanced to visualize (arrow) since GAPDH (loading control) demonstrated less protein in these as compared to the control groups. (B) Full western blots of Pck1 and β-actin as a loading control in liver tissue lysates from Efcab4b+/+ (WT) and Efcab4b−/− (KO). [file mmc1.pptx]
